# Detection of Target Molecules Within One-Millimeter-Thick Mouse Brain Slices by Using Peroxidase-Fused Nanobodies and Fluorochromized Tyramide-Glucose Oxidase Reaction

**DOI:** 10.21769/BioProtoc.5713

**Published:** 2026-06-05

**Authors:** Kenta Yamauchi, Masato Koike, Hiroyuki Hioki

**Affiliations:** 1 Department of Neuroanatomy, Juntendo University Graduate School of Medicine, Tokyo, Japan; 2 Department of Cell Biology and Neuroscience, Juntendo University Graduate School of Medicine, Tokyo, Japan; 3 Department of Multi-Scale Brain Structure Imaging, Juntendo University Graduate School of Medicine, Tokyo, Japan

**Keywords:** FT-GO, Nanobody, Peroxidase, POD-nAb, Three-dimensional immunohistochemistry, Tyramide signal amplification

## Abstract

Three-dimensional immunohistochemistry (3D-IHC) shows the organization of molecular assemblies in the context of tissue architecture. Deep and rapid antibody penetration into 3D tissues and highly sensitive detection are crucial for high-throughput analysis of 3D-IHC imaging. Here, we provide a detailed protocol for a nanobody (nAb)-based 3D-IHC technique, namely POD-nAb/FT-GO 3D-IHC, for high-speed and high-sensitivity detection of targets within 1-mm-thick mouse brain tissues. Peroxidase-fused nAb (POD-nAb) is a genetically encoded recombinant antibody, which consists of a camelid nAb and a variant of horseradish peroxidase, and fluorochromized tyramide-glucose oxidase (FT-GO) is a fluorescent tyramide signal amplification (TSA) system. POD-nAb/FT-GO 3D-IHC incorporates three main components: 1) tissue permeabilization, 2) POD-nAb binding, and 3) 3D-TSA reaction with FT-GO. POD-nAbs enhance signal penetration depth and allow for highly sensitive detection when combined with FT-GO signal amplification. By using the 3D-IHC protocol provided herein, we can visualize target molecules in mouse brain tissues of 1-mm thickness with drastic signal enhancement within three days. This protocol for POD-nAb/FT-GO 3D-IHC could facilitate structural and molecular interrogation of 3D tissues.

Key features

• A high-speed 3D-IHC technique that combines POD-nAbs and FT-GO signal amplification, offering faster results than conventional 3D-IHC using IgG antibodies.

• Visualization of somata and processes of neuronal and glial cells in millimeter-thick mouse brain tissues within three days.

• POD-nAb/FT-GO 3D-IHC offers 9.0- and 6.8-fold signal amplification compared with EGFP fluorescence and 3D-IHC using a synthetic fluorophore-conjugated anti-GFP nAb, respectively.

• POD-nAbs, camelid nanobodies fused with peroxidase, are immunoreagents with high specificity, selectivity, and reproducibility.

## Graphical overview


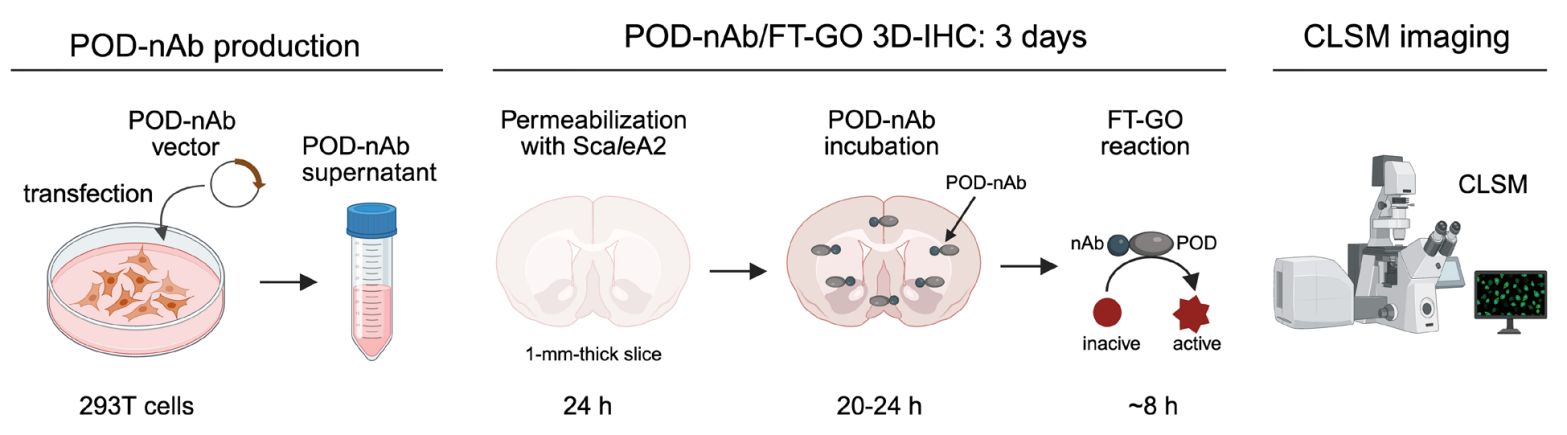


**Overview of POD-nAb/FT-GO 3D-IHC in 1-mm-thick mouse brain slices.** POD-nAbs are produced by transfection of 293T cells with POD-nAb vectors. The culture supernatant is used for 3D-immunolabeling. POD-nAb is a genetically encoded immunoreagent that consists of a camelid nAb and a variant of horseradish peroxidase. POD-nAb/FT-GO 3D-IHC incorporates three main components: (1) tissue permeabilization by incubation with Sca*l*eA2 solution, (2) POD-nAb binding to their antigens, and (3) FT-GO reaction within large-scale tissues. Following the immunolabeling, brain slices are cleared with Sca*l*eS4 solution and imaged by confocal laser scanning microscopy (CLSM). Somata and processes of neurons and glia in a millimeter-thick mouse brain slice are clearly and intensely labeled by the 3D-IHC method within three days. POD-nAb, peroxidase-fused nanobody; 3D-IHC, three-dimensional immunohistochemistry; FT-GO, fluorochromized tyramide-glucose oxidase.

## Background

Three-dimensional immunohistochemistry (3D-IHC), the immunolabeling of 3D tissues, shows molecule–structure–function relationships in a biological system. 3D-IHC provides rich cellular and molecular information to permit reconstruction of microstructural details, unbiased sampling throughout a tissue, and a high data throughput. Recent advances in 3D-IHC techniques have allowed for the visualization of molecular distribution even within human embryos and organs [1–4].

Deep antibody (Ab) penetration in 3D tissues is crucial for staining homogeneity of 3D-IHC. Nanobodies (nAbs), recombinant minimal-antigen-binding fragments from single-chain Abs in camelids [5], should be suitable for 3D-IHC due to their much smaller size (12–15 kDa) than conventional immunoglobulin G (IgG) Abs (~150 kDa). Deep penetration of nAbs has been shown to occur into 3D biological tissues [6–9]. A major issue in the implementation of nAbs to 3D-IHC is signal strength, which imposes a limit on the throughput of 3D-IHC imaging. NAbs conjugated with one or two synthetic fluorophores are typically adopted for 3D-IHC without signal amplification [6–9].

Tyramide signal amplification (TSA) involves the catalytic activity of peroxidase (POD) to yield high-density labeling of targets [10,11], providing a possible solution to the relatively low sensitivity of 3D immunolabeling using nAbs. In our earlier study, we developed a straightforward and cost-effective fluorescent TSA system, fluorochromized tyramide-glucose oxidase (FT-GO) [12]. Unlike conventional TSA systems, FT-GO utilizes hydrogen peroxide (H_2_O_2_) produced by the oxidation of glucose by glucose oxidase to improve the operational stability of the TSA system [12].

Here, we describe a detailed protocol for nAb-based 3D immunolabeling, namely POD-nAb/FT-GO 3D-IHC, for high-speed and high-sensitivity detection of target molecules within 3D tissues. This protocol includes POD-nAb production, 3D-IHC staining with POD-nAbs, 3D-TSA reaction with FT-GO, and confocal laser scanning microscopy (CLSM) of immunolabeled 3D tissues. POD-nAbs (P-RAN-bodies [13]) are camelid nAbs fused with POD. FT-GO reaction within 3D tissues using peroxidase activity of POD-nAbs allows the visualization of target molecules with drastic signal enhancement. GFP POD-nAb/FT-GO 3D-IHC can offer ~10-fold signal amplification compared with EGFP fluorescence [14]. Using the 3D-IHC protocol provided herein, we can visualize somata and processes of neurons and glia in 1-mm-thick mouse brain tissues within three days.

## Materials and reagents

**Biological materials**

1. pCAG-GFP1 POD-nAb-WPRE (1.0 μg/μL) (generated in-house [14], Addgene, #244973)

2. 293T cells (RIKEN BioResource Research Center, RCB2202)

3. AAV2/PHP.eB CAG-EGFP-WPRE (generated in-house [14])

**Caution:** Adeno-associated virus (AAV) is generally classified as a risk group 1 (RG1) virus. AAV vectors can be handled in Biosafety Level 1 (BSL-1) facilities in most cases. However, biosafety should be considered with respect to the precise nature of experiments being conducted.

4. C57BL/6J mice (8–16 weeks old) (Nihon SLC)

**Reagents**

1. Dulbecco’s modified Eagle’s medium (DMEM) (Thermo Fisher Scientific, catalog number: 11965-092)

2. Fetal bovine serum (FBS) (Sigma-Aldrich, catalog number: 173012)

3. L-glutamine (200 mM) (Thermo Fisher Scientific, catalog number: 25030-081)

4. MEM non-essential amino acid (100×) (Thermo Fisher Scientific, catalog number: 11140-050)

5. Penicillin-streptomycin (100×) (Thermo Fisher Scientific, catalog number: 15070-063)

6. Lipofectamine 3000 (Thermo Fisher Scientific, catalog number: L3000001)

7. Opti-MEM I reduced serum medium (Opti-MEM) (Thermo Fisher Scientific, catalog number: 31985062)

8. Thimerosal (Nacalai Tesque, catalog number: 21624-32)

9. Isoflurane (Pfizer)

10. Sodium chloride (NaCl) (Nacalai Tesque, catalog number: 31320-05)

11. Disodium hydrogen phosphate 12-water (Na_2_HPO_4_·12H_2_O) (Nacalai Tesque, catalog number: 31722-45)

12. Potassium chloride (KCl) (Nacalai Tesque, catalog number: 28514-75)

13. Potassium dihydrogenphosphate (KH_2_PO_4_) (Nacalai Tesque, catalog number: 28721-55)

14. Paraformaldehyde (PFA) (Merck Millipore, catalog number: 1.04005.1000)

15. 10 N sodium hydroxide (NaOH) solution (Nacalai Tesque, catalog number: 94611-45)

16. Sodium dihydrogen phosphate dihydrate (NaH_2_PO_4_·2H_2_O) (Nacalai Tesque, catalog number: 31718-15)

17. Pentobarbital sodium salt (Tokyo Chemical Industry, catalog number: P0776)

18. Agar (Nacalai Tesque, catalog number: 01028-85)

19. Sodium azide (NaN_3_) (Nacalai Tesque, catalog number: 31233-55)

20. Triton-X100 (Nacalai Tesque, catalog number: 35501-15)

21. Urea (FUJIFILM Wako Pure Chemical Industries, catalog number: 215-00616)

22. Glycerol (Nacalai Tesque, catalog number: 17018-25)

23. Hydrogen peroxide (H_2_O_2_) (31% aqueous solution) (Santoku Chemical Industries, catalog number: 18412-15)

24. CF tyramide (e.g., CF640R tyramide, Biotium, catalog number: 92175)

25. Dimethyl sulfoxide (DMSO) (FUJIFILM Wako Pure Chemical Industries, catalog number: 043-07216)

26. Glucose oxidase (Nacalai Tesque, catalog number: 16831-14)

27. Bovine serum albumin (BSA) (Nacalai Tesque, catalog number: 01863-77)

28. β-D-glucose (FUJIFILM Wako Pure Chemical Industries, catalog number: 049-31165)

29. D-sorbitol (Nacalai Tesque, catalog number: 06286-55)

30. Agarose (TaKaRa Bio, catalog number: 5003)

**Solutions**

1. 293T cell culture medium (see Recipes)

2. Lipofectamine 3000 solution (see Recipes)

3. Plasmid DNA with P3000 solution (see Recipes)

4. Plasmid DNA–lipid complex (see Recipes)

5. Thimerosal 10% (see Recipes)

6. Phosphate-buffered saline (PBS) (see Recipes)

7. AAV solution (see Recipes)

8. PFA 16% (see Recipes)

9. Phosphate buffer (PB) (pH 7.4) 0.2 M (see Recipes)

10. Fixative solution (see Recipes)

11. Sodium pentobarbital solution (see Recipes)

12. 4% agar in PBS (see Recipes)

13. PB (pH 7.4) 0.1 M (see Recipes)

14. Triton X-100 10% (w/v) (see Recipes)

15. Sca*l*eA2 solution (see Recipes)

16. Endogenous peroxidase quenching solution (see Recipes)

17. Triton X-100 10% (v/v) (see Recipes)

18. PBS-X (see Recipes)

19. POD-nAb solution (see Recipes)

20. CF tyramide solution (see Recipes)

21. GO solution (see Recipes)

22. 2% BSA in 0.1 M PB (see Recipes)

23. FT-GO reaction mixture (see Recipes)

24. β-D-glucose solution (see Recipes)

25. Sca*l*eS4 solution (see Recipes)

26. Sca*l*eS4 D25(0) solution (see Recipes)

27. Sca*l*eS4 gel (see Recipes)

**Recipes**

**1. 293T cell culture medium**

**Table:** 

Reagent	Final concentration	Volume
DMEM	90%	500 mL
FBS	10%	50 mL
L-glutamine	2 mM	5 mL
MEM non-essential amino acid (100×)	1×	5 mL
Penicillin-Streptomycin (100×)	1×	5 mL
Total		565 mL

Swirl the bottle to mix. The medium can be stored at 4 °C for 3 months.

**2. Lipofectamine 3000 solution**

**Table:** 

Reagent	Final concentration	Volume (for 1 well)
Lipofectamine 3000 Reagent	n/a	7.5 μL
Opti-MEM	n/a	125 μL
Total		132.5 μL

Mix well by vortexing for 2–3 s. The solution should be prepared before use. Recipes 2, 3, and 4 are part of the sequential steps for preparing transfection complexes.

**3. Plasmid DNA with P3000 solution**

**Table:** 

Reagent	Final concentration	Volume (for 1 well)
pCAG-GFP1 POD-nAb-WPRE (1.0 μg/μL)	2.5 μg/tube	2.5 μL
P3000 reagent	2 μL/μg DNA	5 μL
Opti-MEM	n/a	125 μL
Total		132.5 μL

Dilute DNA in Opti-MEM and then add P3000 reagent. Mix well by pipetting. The solution should be prepared before use. Recipes 2, 3, and 4 are part of the sequential steps for preparing transfection complexes.

**4. Plasmid DNA–lipid complex**

**Table:** 

Reagent	Final concentration	Volume (for 1 well)
Lipofectamine 3000 solution	50%	125 μL
DNA with P3000 solution	50%	125 μL
Total		250 μL

Mix by tapping the tube. Incubate for 10–15 min at 20–25 °C. The solution should be prepared before use. Recipes 2, 3, and 4 are part of the sequential steps for preparing transfection complexes.

**5. Thimerosal 10%**

**Table:** 

Reagent	Final concentration	Volume (for 10 mL)
Thimerosal	10%	1 g
Double-distilled water (ddH_2_O)	n/a	up to 10 mL
Total		10 mL

Mix well by vortexing. The solution can be stored at 20–25 °C for 1 year.

**Caution:** Thimerosal is an alkylmercury compound. Avoid inhalation or contact with skin, eyes, and mucous membrane. Handle it inside a fume hood with appropriate protective gear.

**Critical:** NaN_3_ as a preservative should be avoided. NaN_3_ inactivates the enzymatic activity of POD.

**6. PBS**

**Table:** 

Reagent	Final concentration	Volume (for 1 L)
NaCl	137 mM	8 g
Na_2_HPO_4_·12H_2_O	8.1 mM	2.9 g
KCl	2.7 mM	200 mg
KH_2_PO_4_	1.5 mM	200 mg
ddH_2_O	n/a	up to 1 L
Total		1 L

Mix well by stirring. The solution can be stored at 20–25 °C for 6 months.

**7. AAV solution**

**Table:** 

Reagent	Final concentration	Volume (for 100 μL)
AAV2/PHP.eB CAG-EGFP-WPRE	5.0 × 10^10^ genome copies/mL	n/a
PBS	n/a	100 μL
Total		100 μL

Mix well by pipetting. The solution should be prepared before use.

**Caution:** Biosafety should be considered with respect to the precise nature of experiments being conducted. AAV vectors can be handled in BSL-1 facilities in most cases.

**Critical:** The optimal concentration of the AAV-GFP vector should be determined for each experiment.

**8. PFA 16%**

**Table:** 

Reagent	Final concentration	Volume (for 1 L)
PFA	16%	160 g
10 N NaOH solution	n/a	n/a
ddH_2_O	n/a	up to 1 L
Total		1 L

Add 160 g of PFA in ddH_2_O at 60–70 °C. Dissolve PFA by adding 10 N NaOH solution and stirring. Allow the solution to cool to 20–25 °C and bring the volume to 1 L with ddH_2_O. Filter the solution through filter paper and dispense to 10 mL each. The solution can be stored at -20 °C for 1 year.

**Caution:** PFA is toxic and teratogenic. Avoid inhalation or contact with skin, eyes, and mucous membranes. Handle it inside a fume hood with appropriate protective gear.

**9. PB (pH 7.4) 0.2 M**

**Table:** 

Reagent	Final concentration	Volume (for 1 L)
NaH_2_PO_4_·2H_2_O	38 mM	11.8 g
Na_2_HPO_4_·12H_2_O	162 mM	116 g
ddH_2_O	n/a	up to 1 L
Total		1 L

Mix well by stirring. The solution can be stored at 20–25 °C for 6 months.

**10. Fixative solution**

**Table:** 

Reagent	Final concentration	Volume (for 40 mL)
PFA 16%	4%	10 mL
PB (pH 7.4) 0.2 M	0.1 M	20 mL
ddH_2_O	n/a	up to 40 mL
Total		40 mL

Filter the solution through filter paper. Mix well by swirling. Use the solution within the same day.

**Caution:** PFA is toxic and teratogenic. Avoid inhalation or contact with skin, eyes, and mucous membranes. Handle it inside a fume hood with appropriate protective gear.

**11. Sodium pentobarbital solution**

**Table:** 

Reagent	Final concentration	Volume (for 20 mL)
Pentobarbital sodium salt	50 mg/mL	1 g
PBS	n/a	up to 20 mL
Total		20 mL

Mix well by stirring. The solution can be stored at 20–25 °C for 1 month.

**12. 4% agar in PBS**

**Table:** 

Reagent	Final concentration	Volume (for 50 mL)
Agar	4%	2 g
PBS	n/a	up to 50 mL
Total		50 mL

Mix well by stirring. Melt agar by heating in a microwave. The solution should be prepared before use.

**13. PB (pH 7.4) 0.1 M**

**Table:** 

Reagent	Final concentration	Volume (for 1 L)
PB (pH 7.4) 0.2 M	0.2 M	500 mL
ddH_2_O	n/a	up to 1 L
Total		1 L

Swirl the bottle to mix. The solution can be stored at 20–25 °C for 3 months.

**14. Triton X-100 10% (w/v)**

**Table:** 

Reagent	Final concentration	Volume (for 50 mL)
Triton X-100	10% (w/v)	5 g
ddH_2_O	n/a	up to 50 mL
Total		50 mL

Mix well by stirring. The solution can be stored at 4 °C for 6 months.

**15. Sca*l*eA2 solution**

**Table:** 

Reagent	Final concentration	Volume (for 100 mL)
Urea	4 M	24.02 g
Glycerol	10% (w/v)	10 g
Triton-X100 10% (w/v)	0.1% (w/v)	1 mL
ddH_2_O	n/a	up to 100 mL
Total		100 mL

Mix well by stirring. The solution can be stored at 4 °C for 1 month.

**16. Endogenous peroxidase quenching solution**

**Table:** 

Reagent	Final concentration	Volume (for 6.2 mL)
H_2_O_2_ 31%	1%	200 μL
PBS	n/a	6 mL
Total		6.2 mL

Mix well by swirling. The solution should be prepared before use.

**Caution:** H_2_O_2_ is corrosive and can cause local tissue damage. Avoid inhalation or contact with skin, eyes, and mucous membranes. Handle it inside a fume hood with appropriate protective gear.

**17. Triton X-100 10% (v/v)**

**Table:** 

Reagent	Final concentration	Volume (for 500 mL)
Triton X-100	10% (v/v)	50 mL
ddH_2_O	n/a	up to 500 mL
Total		500 mL

Mix well by stirring. The solution can be stored at 4 °C for 6 months.

**18. PBS-X**

**Table:** 

Reagent	Final concentration	Volume (for 1 L)
Triton X-100 10% (v/v)	0.3% (v/v)	30 mL
PBS	n/a	up to 1 L
Total		1 L

Swirl the bottle to mix. The solution can be stored at 20–25 °C for 3 months.

**19. POD-nAb solution**

**Table:** 

Reagent	Final concentration	Volume (for 1,200 μL)
POD-nAb culture supernatant	20%	600 μL
Triton X-100 10% (v/v)	0.3% (v/v)	36 μL
Thimerosal 10%	0.02%	2.4 μL
PBS	n/a	up to 1,200 μL
Total		1,200 μL

Mix well by pipetting. The solution should be prepared before use.

**Caution:** Thimerosal is an alkylmercury compound. Avoid inhalation or contact with skin, eyes, and mucous membranes. Handle it inside a fume hood with appropriate protective gear.

**Critical:** The optimal concentration of POD-nAb culture supernatant should be determined for each experiment.

**20. CF tyramide solution**

**Table:** 

Reagent	Final concentration	Volume (for 1 mL)
CF tyramide	2 mM	2 μmol
DMSO	n/a	1 mL
Total		1 mL

Mix well by pipetting. Dispense to 10 μL each. The solution can be stored at -80 °C for 1 year.

**21. GO solution**

**Table:** 

Reagent	Final concentration	Volume (for 1 mL)
Glucose oxidase	1 mg/mL	1 mg
PB (pH 7.4) 0.2 M	0.1 M	500 μL
ddH_2_O	n/a	up to 1 mL
Total		1 mL

Mix well by pipetting. Dispense to 50 μL each. The solution can be stored at -80 °C for 1 year.

**22. 2% BSA in 0.1 M PB**

**Table:** 

Reagent	Final concentration	Volume (for 50 mL)
BSA	2% (w/v)	1 g
PB (pH 7.4) 0.2 M	0.1 M	25 mL
ddH_2_O	n/a	up to 50 mL
Total		50 mL

Mix well by stirring. Sterilize the solution with a 0.22 μm filter. The solution can be stored at 4 °C for 3 months.

**23. FT-GO reaction mixture**

**Table:** 

Reagent	Final concentration	Volume (for 1 mL)
CF tyramide solution	10 μM	5 μL
GO solution	3 μg/mL	3 μL
2% BSA in 0.1 M PB	n/a	992 μL
Total		1 mL

Mix well by pipetting. The solution should be prepared before use.

**24. β-D-glucose solution**

**Table:** 

Reagent	Final concentration	Volume (for 1 mL)
β-D-glucose	200 mg/mL	200 mg
ddH_2_O	n/a	up to 1 mL
Total		1 mL

Mix well by pipetting. Dispense to 50 μL each. The solution can be stored at -80 °C for 1 year.

**25. Sca*l*eS4 solution**

**Table:** 

Reagent	Final concentration	Volume (for 100 mL)
Urea	4 M	24.02 g
D-sorbitol	40% (w/v)	40 g
Glycerol	10% (w/v)	10 g
Triton-X100 10% (w/v)	0.2% (w/v)	2 mL
DMSO	25% (v/v)	25 mL
ddH_2_O	n/a	up to 100 mL
Total		100 mL

For a detailed procedure for preparation of this solution, refer to Miyawaki et al. [15]. The solution can be stored at 4 °C for 1 month.

**26. Sca*l*eS4 D25(0) solution**

**Table:** 

Reagent	Final concentration	Volume (for 100 mL)
Urea	4 M	24.02 g
D-sorbitol	40% (w/v)	40 g
Glycerol	10% (w/v)	10 g
DMSO	25% (v/v)	25 mL
ddH_2_O	n/a	up to 100 mL
Total		100 mL

For a detailed procedure for preparation of this solution, refer to Miyawaki et al. [15]. The solution can be stored at 4 °C for 1 month.

**27. Sca*l*eS4 gel**

**Table:** 

Reagent	Final concentration	Volume (for 100 mL)
Agarose	1.5% (w/v)	1.5 g
Sca*l*eS4 D25(0)	n/a	up to 100 mL
Total		100 mL

For a detailed procedure for preparation of this solution, refer to Miyawaki et al. [15]. The solution can be stored at 4 °C for 1 year. Sca*l*eS4 gel solidifies at 4 °C. Remelt the gel by heating it in a microwave before use.

**Laboratory supplies**

1. 50 mL conical tubes (Greiner Bio-One, catalog number: 227651)

2. Syringe filter (0.22 μm) (Merck Millipore, catalog number: SLGPR33RB)

3. Filter paper No. 2 (ADVANTEC, catalog number: 00021240)

4. 30-G insulin syringe (NIPRO, catalog number: 08-277)

5. 23-G regular bevel needle (Terumo Corporation, catalog number: NN-2332R)

6. 20 mL syringe (Terumo Corporation, catalog number: SS-20ESZ)

7. Surgical scissors (FRIGZ, catalog number: E221-288)

8. Tweezers (FRIGZ, catalog number: E380-546, E502-004)

9. 15 mL conical tubes (Greiner Bio-One, catalog number: 188271-N)

10. Cell culture multi-well plates, 6 well (6-well plate) (Greiner Bio-One, catalog number: 657160)

11. Razor blade (Feather Safety Razor, catalog number: FAS-10)

12. Superglue (Aron Alpha^®^) (Toagosei, catalog number: 201)

13. Micro spatula, stainless steel (AS ONE, catalog number: 9-891-03)

14. Safe-lock tubes, 2.0 mL (Eppendorf, catalog number: 0030120094)

15. Custom-made imaging chambers [16]; three-dimensional computer-aided design (3D CAD) data for the imaging chamber are provided in Furuta et al. [16]

16. Coverslips (24 × 40 mm) (Matsunami Glass, catalog number: C024401)

17. Kimwipe (NIPPON PAPER CRECIA, catalog number: S-200)

18. Slide glasses (26 × 76 mm) (Matsunami Glass, catalog number: S1225)

19. Metal weights (Hikari, catalog number: GZ25)

20. Glass petri dish (60 mm) (SANSYO, catalog number: 82-1683)

21. Blu-Tack^®^ (Bostik, catalog number: CKBT-450000)

## Equipment

1. Clean bench (Thermo Scientific, model: 1300 Series A2)

2. CO_2_ incubator (Thermo Fisher Scientific, model: Forma Steri-Cycle 370)

3. Centrifuge with rotors for 15 and 50 mL tubes (Tomy, model: MDX-310)

4. Fume hood (Dalton, model: Uni-optflow DFA10-AA15-AA10)

5. Rotary shaker (TAITEC, model: NR-20)

6. Vibratome (Dosaka EM, model: PRO7N)

7. Constant temperature incubator shaker (TAITEC, model: BR-23FP)

8. Confocal scanning microscope system (Leica Microsystems, model: TCS SP8)

9. 16× multi-immersion objective lens (Leica Microsystems, model: HC FLUOTAR 16×/0.60 IMM CORR VISIR)

## Software and datasets

1. LAS-X (Leica Microsystems, v3.5.5.19976, February 2021)

2. Fiji (v2.16.0/1.54p, October, 2024)

*Note: LAS-X software requires a license.*

## Procedure

**A. Production of POD-nAb**

*Note: Perform steps A1–3 inside a clean bench.*

1. Plate 293T cells on a 6-well plate in 2 mL per well of 293T cell culture medium so they will be 70%–90% confluent on the day of transfection.

2. Prepare Lipofectamine 3000 solution, POD-nAb plasmid DNA with P3000 solution, and then plasmid DNA–lipid complex (see Recipes 2–4).

3. Add 250 μL per well of the plasmid DNA–lipid complex to the 293T cells.

4. Incubate the cells for 3 days at 37 °C in a CO_2_ incubator (5% CO_2_, 95% humidity).

5. Collect the culture supernatant into a 50 mL conical tube.

6. Remove cell debris by centrifugation at 3,000× *g* for 5 min at 4 °C. Filter the culture supernatant through a 0.22 μm syringe filter. Add thimerosal 10% into the POD-nAb culture supernatant at the final concentration of 0.05% and store at 4 °C. The POD-nAb culture supernatant can be stored at 4 °C for 6 months and used for immunolabeling.

**Caution:** Thimerosal is an alkylmercury compound. Avoid inhalation or contact with skin, eyes, and mucous membranes. Handle it inside a fume hood with appropriate protective gear.

**B. AAV vector injection**

**Caution:** Biosafety should be considered with respect to the precise nature of experiments being performed. AAV vectors can be handled in BSL-1 facilities in most cases.

1. Anesthetize an adult mouse (8–16 weeks old) by isoflurane inhalation.

2. Inject 100 μL of an AAV solution into the retro-orbital sinus of the mouse using a 30-G insulin syringe.

**Critical:** The optimal concentration of the AAV-GFP vector should be determined for each experiment.

3. Return the mouse to its home cage with ad libitum access to food and water. House the mouse in specific pathogen-free conditions for 3–4 weeks. Monitor the health and behavior of the mouse at least twice a week.

**C. Tissue preparation**

**Figure 1.**
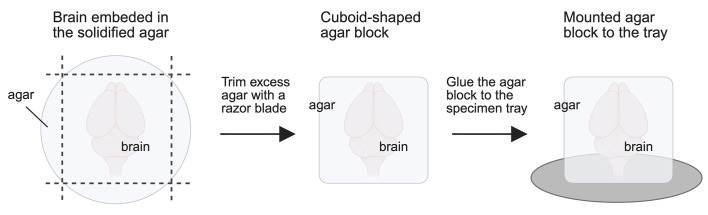
Preparation of agar blocks for tissue slicing with a vibratome. An embedded brain tissue and agar are removed from a well of a 6-well plate. Excess agar around the brain tissue is trimmed away to form a cuboid shape using a razor blade. The brain tissue should be kept in the middle of the agar block. The cuboid agar block is mounted onto the bottom of a specimen tray with super glue.

Following perfusion fixation and post-fixation, brain tissues infected with the AAV-GFP vector are cut into 1-mm-thick brain slices using a vibratome. Preparation of agar blocks for tissue slicing with a vibratome is shown in Figure 1.

**Caution:** PFA is toxic and teratogenic. Avoid inhalation or contact with skin, eyes, and mucous membranes. Perform steps C1–6 inside a fume hood with appropriate protective gear to limit the exposure to PFA.

1. Filter PBS and fixative solution through filter paper and chill on ice.

2. Anesthetize the mouse with an intraperitoneal injection of sodium pentobarbital solution (200 mg/kg). Assess the depth of anesthesia by eye-blink reflexes and toe-pinch withdrawal.

3. Open the thoracic cavity using surgical scissors, cut the right atrial appendage, and insert a 23-G needle into the left ventricle of the heart from the apex.

4. Perfuse with 20 mL of ice-cold PBS using a 20 mL syringe over 2–3 min.

5. Perfuse with 20 mL of the ice-cold fixative with another 20 mL syringe over 2–3 min.

6. Excise the brain from the skull using surgical scissors and tweezers.

7. Transfer the brain in 12 mL of fixative solution in a 15 mL conical tube and gently shake (50–100 rpm) for 24 h at 4 °C.

**Pause point:** Brain tissues can be stored in 0.02% NaN_3 _in PBS at 4 °C for 4 weeks. Brain tissues should be washed extensively with PBS after storage to remove NaN_3_.

8. Pour 10 mL of 4% agar in PBS into a well of a 6-well plate.

9. Submerge the brain tissue in the agar solution and leave the plate on ice until the agar solidifies.

*Note: The temperature of the agar solution should be between 40 and 45 °C.*

10. Remove the embedded brain tissue and agar from the well and trim excess agar around the brain tissue to form a cuboid shape using a razor blade. Keep the brain tissue in the middle of the agar block.

11. Glue the agar block to the bottom of a specimen tray with super glue and pour PB (pH 7.4) 0.1 M into the bath.

12. Cut 1-mm-thick brain slices using a vibratome and collect the slices in 8 mL of PBS in a well of a 6-well plate.

**Pause point:** Brain slices can be stored in 0.02% NaN_3_ in PBS at 4 °C for 4 weeks. Brain slices should be washed extensively with PBS after storage to remove NaN_3_.

**D. 3D-immunolabeling with POD-nAb/FT-GO**

**Critical:** Brain slices should be shaken thoroughly in solutions during the staining procedure. This is critical for staining homogeneity and the signal/noise ratio in POD-nAb/FT-GO 3D-IHC.

**Critical:** From step D8 onward, brain slices should be protected from light to avoid fading of the fluorescent dye.

1. Transfer 2–3 brain slices with a spatula into 8 mL of prewarmed Sca*l*eA2 solution in a 6-well cell culture plate and incubate for 24 h at 37 °C with shaking (90 rpm) on a constant-temperature incubator shaker.

2. Wash the brain slices with 8 mL of PBS in a 6-well cell culture plate for 15 min twice on a rotary shaker at 20–25 °C with gentle rocking (50–100 rpm).

**Pause point:** Brain slices can be stored in 0.02% NaN_3_ in PBS at 4 °C for 1 week. Brain slices should be washed extensively with PBS after storage to remove NaN_3_.

3. Transfer the brain slices with a spatula into 6 mL of endogenous peroxidase quenching solution in a 6-well cell culture plate and incubate for 1 h on the rotary shaker at 20–25 °C with gentle rocking (50–100 rpm).

**Caution:** H_2_O_2_ in the endogenous peroxidase quenching solution is corrosive and can cause local tissue damage. Avoid inhalation or contact with skin, eyes, and mucous membranes. Handle the quenching solution inside a fume hood with appropriate protective gear.

**Critical:** Inadequate blocking of endogenous peroxidase leads to false-positive signals.

4. Wash the brain slices with 8 mL of PBS in a 6-well cell culture plate for 15 min twice on the rotary shaker at 20–25 °C with gentle rocking (50–100 rpm).

5. Transfer the brain slices into 1,200 μL of POD-nAb solution in a 2 mL safe-lock tube with a spatula. Incubate the brain slices for 20–24 h on the rotary shaker at 20–25 °C with gentle rocking (50–100 rpm).

6. Wash the brain slices with PBS-X in a 6-well cell culture plate for 30 min four times on the rotary shaker at 20–25 °C with gentle rocking (50–100 rpm).

7. Wash the brain slices with PB (pH 7.4) 0.1 M in a 6-well cell culture plate for 5 min three times on the rotary shaker at 20–25 °C with gentle rocking (50–100 rpm).

8. Transfer the brain slices into 1,200 μL of FT-GO reaction mixture in a 2 mL safe-lock tube with a spatula and incubate the brain slices for 4 h on the rotary shaker at 20–25 °C with gentle rocking (50–100 rpm).

9. Add 12 μL of β-D-glucose solution into the FT-GO reaction mixture and incubate the brain slices for 30–120 min on the rotary shaker at 20–25 °C with gentle rocking (50–100 rpm).

**Critical:** The optimal duration of the FT-GO reaction should be determined for each experiment. The optimal duration can be determined by whether signal enhancement has reached saturation.

10. Wash the brain slices with PBS-X in a 6-well cell culture plate for 15 min twice on the rotary shaker at 20–25 °C with gentle rocking (50–100 rpm).

11. With a spatula, transfer the brain slices into 6 mL of fixative solution in a 6-well cell culture plate. Incubate for 20–24 h on the rotary shaker at 4 °C with gentle rocking (50–100 rpm).

**Caution:** PFA in the fixative solution is toxic and teratogenic. Avoid inhalation or contact with skin, eyes, and mucous membranes. Handle the solution inside a fume hood with appropriate protective gear.

12. Wash the brain slices with 8 mL of PBS in a 6-well cell culture plate for 15 min twice on the rotary shaker at 20–25 °C with gentle rocking (50–100 rpm).

**Pause point:** Brain slices can be stored in 0.02% NaN_3_ in PBS at 4 °C for 4 weeks.

**E. Tissue clearing and CLSM imaging**

**Figure 2.**
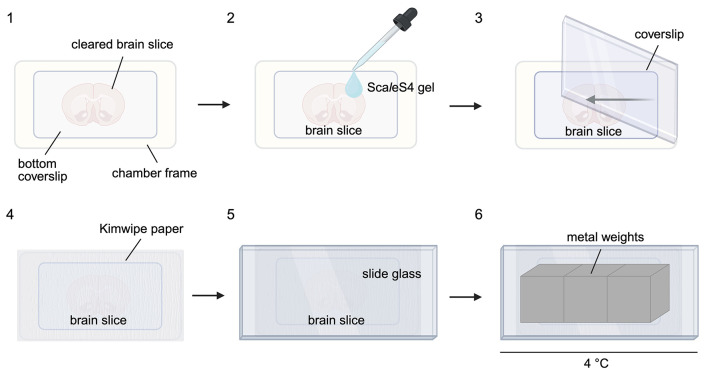
Mounting brain slices on the imaging chamber. Customizable 3D-printed imaging chambers are used for mounting of cleared brain slices. 3D CAD data for the imaging chamber are provided in Furuta et al. [16]. The imaging chamber is composed of a chamber frame and a bottom coverslip [16]. The procedure for mounting brain slices is as follows: 1) mount cleared brain slices on the bottom coverslip of an imaging chamber; 2) drop Sca*l*eS4 gel on the cleared brain slice to fill the imaging chamber; 3) mount a coverslip on the imaging chamber filled with Sca*l*eS4 gel; 4) place a piece of Kimwipe paper on the coverslip; 5) place a glass slide on top of the paper; and 6) place metal weights on the slide glass in a refrigerator at 4 °C. Excessive Sca*l*eS4 solution around the cleared brain slices should be removed before dropping Sca*l*eS4 gel.

*Note: We use a customizable 3D-printed imaging chamber for mounting cleared brain slices (Figure 2). 3D CAD data for the imaging chamber are provided in Furuta et al. [16]. Detailed procedures for tissue mounting onto the imaging chamber are also described in Yamauchi et al. [17,18].*

1. Transfer the brain slices with a spatula into 8 mL of prewarmed Sca*l*eS4 solution in a 6-well cell culture plate and incubate for 12–16 h at 37 °C with shaking (90 rpm) on the constant-temperature incubator shaker. The brain slices become transparent after incubation in Sca*l*eS4 solution.

2. Place a cleared brain slice on the bottom coverslip of an imaging chamber.

3. Remove excessive Sca*l*eS4 solution around the cleared brain slice.

4. Drop 600–800 μL of Sca*l*eS4 gel on the cleared slice to fill the imaging chamber.

5. Mount a coverslip on the imaging chamber filled with Sca*l*eS4 gel. Place a piece of Kimwipe paper on the coverslip and then place a glass slide on top of the paper.

6. Transfer the imaging chamber to a refrigerator at 4 °C.

7. Place metal weights on the slide glass and allow Sca*l*eS4 gel to solidify for 30–60 min.

8. Remove the metal weights, slide glass, and Kimwipe paper from the imaging chamber and wipe away excess Sca*l*eS4 gel.

9. Attach the imaging chamber to the bottom of a 60-mm glass Petri dish with Blu-Tack^®^. Adhesion at multiple points is required. For a detailed procedure for attachment of the imaging chamber, refer to Yamauchi et al. [17,18].

10. Pour Sca*l*eS4 solution into the dish and incubate the imaging chamber containing the cleared brain slice in the solution for 30–60 min.

11. Substitute with fresh Sca*l*eS4 solution.

12. Set the correction collar of a 16× multi-immersion objective lens to 1.47.

**Critical:** Refractive index (RI) mismatch-induced aberrations can disturb image formation [17]. The Sca*l*eS4 solution has an RI of approximately 1.47 [15,19].

13. Mount the 60-mm glass Petri dish containing the imaging chamber on a microscope stage.

14. Immerse the objective lens in Sca*l*eS4 solution and make it slowly approach the cleared brain slice.

**Critical:** Air bubbles trapped on the tip of the objective lens should be removed.

15. Adjust imaging acquisition settings. These include laser power, scan speed, pinhole diameter, detector gain, amplifier offset/gain, *xy* and *z*-axis resolution, and bit intensity resolution. Images should be acquired using a z-compensation mode in which the laser intensity and/or detection gain increase with imaging depth to compensate for depth-dependent signal loss.

16. Collect images by CLSM and save the acquired images.

17. Image processing is done with LAS-X and/or Fiji software.

## Validation of protocol

This protocol or parts of it have been used and validated in the following research article:

• Yamauchi et al. [14]. A three dimensional immunolabeling method with peroxidase-fused nanobodies and fluorochromized tyramide-glucose oxidase signal amplification. Commun Biol (Figures 1c, 5b–c, and 8c–d, and Supplementary Movies 1 and 3).

**Figure 3.**
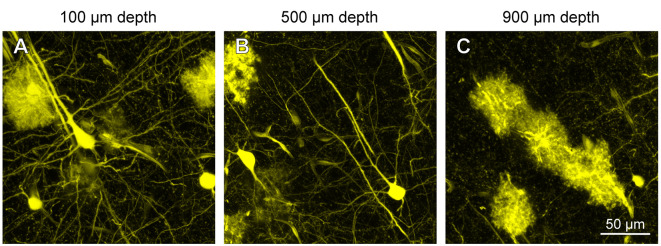
GFP POD-nAb/FT-GO 3D-IHC in a 1-mm-thick mouse brain slice. (A–C) *xy* images at depths of 100, 500, and 900 μm in a 1-mm-thick brain slice stained by POD-nAb/FT-GO 3D-IHC for GFP. The brain slice is prepared from a mouse infected with AAV2/PHP.eB CAG-EGFP-WPRE. The images are acquired with z-compensation to compensate for depth-dependent signal loss. The pinhole size is set to 1.0 Airy unit during imaging acquisition. Scale bar: 50 μm. POD-nAb, peroxidase-fused nanobody; FT-GO, fluorochromized tyramide-glucose oxidase; 3D-IHC, three-dimensional immunohistochemistry; GFP, green fluorescent protein.

We provide validation data in Figure 3, which shows an example of GFP POD-nAb/FT-GO 3D-IHC in the cerebral cortex of a 1-mm-thick brain slice infected with AAV2/PHP.eB CAG EGFP WPRE (n = 3 slices from 3 mice). *xy* images obtained at depths of 100, 500, and 900 μm are represented in each panel (Figure 3A–C). Neuronal and glial processes in a 1-mm-thick mouse brain slice are stained with a high signal/noise ratio by POD-nAb/FT-GO 3D-IHC for GFP. The staining procedure of GFP POD-nAb/3D-IHC can be completed within three days. POD-nAb/FT-GO 3D-IHC allows for highly sensitive detection of target molecules in mouse brain tissues. We have shown that POD-nAb/FT-GO for GFP yields 9.0- and 6.8-fold signal amplification compared with EGFP fluorescence and conventional 3D-IHC using a synthetic fluorophore-conjugated anti-GFP nAb, respectively [14].

## General notes and troubleshooting

**General notes**

Adequate fixation is essential for POD-nAb/FT-GO 3D-IHC. While overfixation can cause denaturation or modification of antigens to increase background fluorescence and shrinkage of tissue samples, inadequate fixation can lead to loss of antigens and degradation of tissue integrity. Blocking endogenous POD activity by incubation with H_2_O_2_ is crucial to prevent false positives in POD-nAb/FT-GO 3D-IHC. NaN_3_ inactivates the enzymatic activity of POD. NaN_3_ as a preservative should be avoided for the POD-nAb supernatant and solution. NaN_3_ should be completely washed out from brain tissues following prolonged storage. Residual NaN_3_ can inactivate POD fused with nAbs to compromise FT deposition onto tissues. The tissue permeabilization with Sca*l*eA2 solution can be omitted from the 3D-IHC protocol for a POD-nAb [14].

Our current POD-nAb/FT-GO 3D-IHC protocol is applicable to at least three proteins, including an endogenous protein, ITGAM. We further implemented multiplex labeling to POD-nAb/FT-GO 3D-IHC by quenching POD with NaN_3_ [14]. However, currently, POD-nAbs available for 3D-IHC using FT-GO reaction still remain quite scarce. A major limitation of the protocol is the scaling of the 3D-IHC. The applicability of our current POD-nAb/FT-GO 3D-IHC protocol is limited to mouse brain tissues of 1-mm thickness [14]. Spatial resolution is one of the main considerations for our current 3D-IHC protocol. TSA, including FT-GO reaction, can lead to blurring of signals owing to the free diffusion of tyramide radicals before their covalent deposition. Addition of viscosity-increasing agents and/or an agent that reduces the lifetime of tyramide radicals [20,21] into FT-GO reaction mixtures might reduce the signal blurring of POD-nAb/FT-GO 3D-IHC.

**Troubleshooting**

**Problem 1:** Absent immunosignal.

Possible causes: Absent POD-nAb production in cultures or inactivation of POD fused with nAbs.

Solution: Prepare fresh solutions and reagents for plasmid transfection. NaN_3_ as a preservative should be avoided for the POD-nAb supernatant and solution.

**Problem 2:** Weak immunosignal.

Possible causes: Very high or low concentrations of POD-nAb in a reaction solution.

Solution: Adjust the concentration of POD-nAb in a reaction solution by increasing or decreasing the POD-nAb culture supernatant. Whether the issue is caused by very high or very low concentration cannot be determined.

**Problem 3:** High background.

Possible causes: High concentrations of POD-nAb in a reaction solution and/or insufficient washes of brain tissues.

Solutions: Decrease the concentration of POD-nAb in a reaction solution and/or increase the number of washes to remove residual POD-nAb.

**Problem 4:** False positive signals.

Possible cause: Insufficient inactivation of endogenous peroxidase.

Solutions: Consider extending the incubation time of the endogenous peroxidase quenching solution and/or increase the concentration of H_2_O_2_ in the endogenous peroxidase quenching solution.

**Problem 5:** Limited signal penetration depth.

Possible cause: Limited penetration of POD-nAbs into brain tissues.

Solution: Extend the incubation time of the antibody solution and/or increase the concentration of POD-nAb. Brain tissues should be shaken thoroughly for deep penetration of POD-nAb. Prolonged incubation time in Sca*l*eA2 solution can enhance tissue permeability.

**Problem 6:** Insufficient clearing of brain tissues.

Possible causes: Denaturing of Sca*l*eS4 solution and/or inadequate incubation.

Solutions: Use fresh Sca*l*eS4 solution. Sca*l*eS4 solution can be stored for up to 1 month at 4 °C. Consider prolonged incubation in Sca*l*eS4 solution.
